# Supplementary material for: Knee Kinetics and Kinematics of Young Asymptomatic Participants during Single-Leg Weight-Bearing Tasks: Task and Sex Comparison of a Cross-Sectional Study
Source: Int J Environ Res Public Health. 2022 May 4;19(9):5590. doi: 10.3390/ijerph19095590 (PMC9104880; doi:10.3390/ijerph19095590)
Supplement: Supplementary file 1 [file ijerph-19-05590-s001.zip › ijerph-1688888-supplementary.pdf]

Supplementary Table S1. Knee kinetics and kinematics during single-leg weight-bearing tasks of asymptomatic young people (n = 30): median and interquartile range in the moment of occurrence of the articular peak moment and the angular peak of the knee in both the frontal and sagittal plane during stair descent, single-leg step down and single-leg squat.

		Stair Descent	Single-leg step down	Single-leg squat
Frontal Plane	**Articular moment peak of the knee**			
	Articular moment (Nm/kg)	-0.88 (0.49)	-1.00 (0.53)	-2.37 (1.21) * †
	Angle (°)	+1.44 (5.44)	+3.39 (8.06)*	+ 2.09 (6.47)*
	**Angular peak of the knee**			
	Articular moment (Nm/kg)	-0.15 (0.64) †	-0.87 (0.71) *	-1.91 (1.07) *†
	Angle (°)	+3.71 (11.72)	+5.70 (7.30) *	+4.18 (11.16)
Sagittal Plane	**Articular moment peak of the knee**			
	Articular moment (Nm/kg)	-2.15 (0.78)	-1.63 (1.26)	-2.07 (4.66)
	Angle (°)	+54.17 (18.94) †	+67.96 (12.19)	+56.56 (61.70)†
	**Angular peak of the knee**			
	Articular moment (Nm/kg)	+0.09 (0.09)	-1.40 (1.25) *	-2.03 (2.38) *
	Angle (°)	+86.72 (7.10)	+75.46 (14.38)	+67.14 (12.16)*†

Note: Symbols represent in the frontal plane abduction / varus (+) and adduction / valgus (-) of the knee, and in the sagittal plane, flexion (+) extension (-) of the knee.* Different stair descent (p <0.05).† Different from single-leg step down (p <0.05).

Supplementary Table S2. Kinetics and kinematics of the knee stratified by sex: median and interquartile range of males (n = 15) and females (n = 15) in the single-leg weight-bearing tasks evaluated at the articular moment peak and angular peak of the knee.

		Knee joint moment peak frame				Knee peak angle frame			
		Frontal Plane		Sagittal Plane		Frontal Plane		Sagittal Plane	
		Articular moment (Nm/kg)	Angle (°)	Articular moment (Nm/kg)	Angle (°)	Angle (°)	Articular moment (Nm/kg)	Angle (°)	Articular moment (Nm/kg)
**Stair descent**	**Male**	-0.81 (0.55)	+1.97 (4.47)	-2.09 (0.41)	+59.78 (17.82)	+3.10 (7.16)	-0.18 (0.59)	+86.82 (8.55)	+0.07 (0.10)
	**Female**	-0.91 (0.40)	-0.24 (8.04)	-2.17 (0.99)	+49.22 (15.05)	+3.95 (15.00)	-0.01 (0.60)	+85.84 (7.14)	+0.10 (0.07)
**Single-leg step down**	**Male**	-0.91 (0.52)	+3.46 (8.25)	-1.72 (1.13)	+66.34 (14.31)	+8.19 (7.85)	-0.60 (0.55)	+82.27 (14.98)	-1.50 (1.11)
	**Female**	-1.11 (0.48)	+2.55 (6.79)	-1.28 (0.20)	+68.84 (13.57)	+4.15 (11.84)	-1.02 (0.50)	+74.02 (12.38)	-1.25 (0.97)
**Single-leg squat**	**Male**	-2.42 (1.09)	+3.18‡ (6.77)	-2.54 (4.37)	+60.85 (45.31)	+4.23 (5.59)	-2.11 (0.93)	+66.10 (13.06)	-2.48 (4.22)
	**Female**	-2.33 (1.21)	+0.08‡ (5.25)	-1.78 (4.19)	+50.38 (57.91)	+4.12 (13.02)	-1.90 (1.04)	+68.18 (9.48)	-1.78 (1.42)

Note: Symbols represent knee abduction / varus (+) and knee adduction / valgus (-), and in the sagittal plane, (+) knee extension and (-) flexion. ‡ Sex difference (p <0.05).

Supplementary Table S3. Adjacent joints: kinetic and kinematic data of the adjacent joints at the moment of peak knee joint moment of the total sample (n = 30) and stratified by sex (male, n = 15; female, n = 15).

		Hip				Ankle			
		Frontal Plane		Sagittal Plane		Frontal Plane		Sagittal Plane	
		Articular moment (Nm/kg)	Angle (°)	Articular moment (Nm/kg)	Angle (°)	Articular moment (Nm/kg)	Angle (°)	Articular moment (Nm/kg)	- Angle (°)
**Stair descent**	**Total**	+0.98 (0.65)	-0.86 (5.67)	+2.66 (1.27)	+4.99 (10.98)	-0.34 (0.54)	-12.74 (5.22)	-0.32 (0.46)	+2.94 (13.37)
	**Male**	+1.18 (0.74)	+2.41‡ (5.36)	+2.52 (0.84)	+7.69 (11.84)	-0.28 (0.62)	-12.67 (4.79)	-0.12 (0.43)	+0.71 (8.61)
	**Female**	+0.92 (0.56)	-2.27‡ (4.62)	+2.76 (1.28)	+2.95 (7.70)	-0.43 (0.52)	-13.85 (6.46)	-0.40 (0.38)	+8.06 (10.46)
**Single-leg step down**	**Total**	+1.29 (0.65)	-9.98* (9.24)	+0.28* (1.00)	+45.41* (19.52)	-0.42 (0.51)	-5.07* (4.87)	-0.38 (0.85)	+10.11* (11.77)
	**Male**	+1.16 (0.48)	-8.51 (8.60)	+0.61 (1.41)	+47.01 (15.95)	-0.36 (0.44)	-5.24 (4.90)	-0.12 (1.12)	+5.38 (11.76)
	**Female**	+1.43 (0.77)	-10.90 (11.29)	+0.22 (0.72)	+42.05 (19.55)	-0.50 (0.51)	-5.07 (3.13)	-0.64 (0.59)	+11.04 (7.59)
**Single-leg squat**	**Total**	+2.75*† (0.93)	-5.33* (8.81)	+0.61* (2.36)	+39.74* (44.31)	-0.51 (0.74)	-5.60* (3.22)	-0.48 (2.45)	+4.76 (26.44)
	**Male**	+2.64 (0.81)	-3.68 (6.74)	+0.86 (3.71)	+46.19 (26.04)	-0.48 (0.31)	-5.70 (2.50)	-0.16 ‡ (3.55)	+4.02 (17.93)
	**Female**	+2.79 (1.13)	-6.36 (6.11)	+0.20 (2.01)	+34.46 (37.03)	-0.70 (0.79)	-5.51 (4.28)	-0.59‡ (1.29)	+5.51 (28.01)

* Note: Symbols represent in the frontal plane abduction (+) and adduction (-) for hip and ankle, and in the sagittal plane, hip flexion (+), extension (-) or dorsiflexion (+) and plantar flexion (-) of ankle.* Different stair descent (p <0.05) † Different from single-leg step down (p <0.05) ‡ Sex difference (p <0.05).
